# Characteristics and correlates of seclusion and mechanical restraint measures in a Parisian psychiatric hospital group

**DOI:** 10.3389/fpsyt.2024.1296356

**Published:** 2024-02-19

**Authors:** Valerie Dauriac-Le Masson, Fabienne El-Khoury Lesueur, Justine Lahaye, Corinne Launay, Alexandre Christodoulou, Catherine Boiteux, Judith Maman, Xavier Bonnemaison, Florence Perquier, Marie-Noelle Vacheron

**Affiliations:** ^1^Département d’information Medicale, GHU Paris Psychiatrie et Neurosciences, Paris, France; ^2^Cellule épidémiologie, GHU Paris Psychiatrie et Neurosciences, Paris, France; ^3^Sorbonne Universite, INSERM UMRS_1136, Institut Pierre Louis d’Epidémiologie et de Santé Publique IPLESP, Paris, France; ^4^Pôle Psychiatrie Précarité, GHU Paris Psychiatrie et Neurosciences, Paris, France; ^5^Pôle 75G05-06, GHU Paris Psychiatrie et Neurosciences, Paris, France; ^6^Pôle 75G28, GHU Paris Psychiatrie et Neurosciences, Paris, France; ^7^Esquirol Hospital, Saint-Maurice, France; ^8^Mental Health Association of the 13th (ASM13), Paris, France; ^9^Pôle 14, GHU Paris Psychiatrie et Neurosciences, Paris, France

**Keywords:** seclusion, restraint, prevention, organization of care, duration of seclusion, predictive factors, psychiatric inpatients

## Abstract

**Introduction:**

Seclusion or restraint (S/R) are last-resort measures used in psychiatry to ensure the safety of the patient and the staff. However, they have harmful physical and psychological effects on patients, and efforts to limit their use are needed. We describe the characteristics and correlates of S/R events in four Parisian psychiatric centers.

**Methods:**

Within a 3-month period, November 5, 2018 to February 3, 2019, we recorded data for patients experiencing an S/R measure as well as characteristics of the measures. We studied the mean duration of a S/R event, the time between hospital admission and the occurrence of the event, as well as correlates of these durations. We also examined factors associated with use of a restraint versus a seclusion measure.

**Results:**

For the 233 patients included, we recorded 217 seclusion measures and 64 mechanical restraints. Seclusion measures mostly occurred after the patient’s transfer from the emergency department. The duration of a seclusion measure was about 10 days. Patients considered resistant to psychotropic treatments more frequently had a longer seclusion duration than others. The mean duration of a mechanical restraint measure was 4 days. Male sex and younger age were associated with experiencing mechanical restraint.

**Discussion:**

S/R measures mostly occur among patients perceived as resistant to psychotropic drugs who are arriving from the emergency department. Developing specific emergency department protocols might be useful in limiting the use of coercive measures.

## 1 Introduction

Seclusion is the involuntary confinement of a patient who is alone in a space that they are physically prevented from leaving. Restraint is the involuntary partial or fully immobilization of a patient. These two procedures (seclusion/restraint [S/R]) are last-resort measures used in psychiatry for managing violent behavior such as auto-destructive and suicidal behaviors and aggressive behavior toward other patients and/or staff. They are also used to prevent escaping and for other acute behavioral disturbances (1). Therefore, these measures mostly serve to ensure the safety of patients and adequate treatment.

However, these measures are known to have harmful physical and psychological effects on patients, such as revival of previous trauma, deep vein thrombosis, increased length of stay, and negative emotions (2). They also are an infringement on patients’ autonomy and dignity (3). Therefore, the Council of Europe recommends reducing as much as possible the resort to restraints as well as decreasing their duration (4). In France, the directive of the healthcare system Modernization Act of January 26, 2016 and Article 84 of the law of December 14, 2020 on the financing of social security for 2021 follow the same lines: S/R is indicated when “strictly necessary” in general psychiatry, after patient evaluation, and must be considered as a last resort (5–7).

There have been numerous studies worldwide on the use of S/R measures for psychiatric patients as well as programs to reduce such coercive measures (8, 9). Studies report significant heterogeneity in the frequency and duration of these measures between countries (10, 11) and between mental health services within the same institution. Although there are recent and detailed data on the duration of these measures (12–15), the reasons for and modalities of restraint or their potential adverse effects, as well as the characteristics of the patients, are relatively scarce, as many studies rely on the analysis of registries. However, more precise data are needed to better understand the potential risk factors for the use of isolation, restraint and their respective durations in order to address these factors and ultimately reduce the incidence of coercive measures in psychiatry.

In this study, we examined the characteristics and correlates of S/R events in the four general psychiatric establishments of the Paris metropolitan area.

## 2 Methods

### 2.1 Population

All adults undergoing treatment at the full-time psychiatric hospitalization units in Parisian psychiatric wards, not including those receiving treatment solely in emergency services, were eligible for the study. Patients were recruited if they were had had a seclusion and/or mechanical restraint measure and were hospitalized from November 5, 2018 to February 3, 2019 and if they or their legal guardian did not oppose study participation. Informed consent was obtained from all participants and/or their legal guardian(s).

### 2.2 Study centers

Participants were included from four different Parisian psychiatric centers: Centre Hospitalier Sainte Anne (7 departments, 332 beds), Maison Blanche (22 departments, 496 beds), Hopitaux de Saint-Maurice (5 departments, 97 beds) and Association de Santé Mentale du 13e arrondissement de Paris (3 departments, 49 beds). These establishments serve all sectors of general psychiatry in the Paris healthcare area: admissions are according to the principle of sectorization (i.e., each ward is assigned to a specific sector of the catchment area). Altogether, these four establishments have a catchment area of 1.8 million Parisians.

### 2.3 Data collection

The prospective cohort study was performed under real care conditions with no study-related interventions. All treatments were according to standard procedures in the respective hospitals.

#### 2.3.1 Socio-demographic characteristics

A nurse collected data on each patient’s sex, age, mode of commitment in psychiatric care (voluntary/involuntary), and housing condition (experiencing homelessness/at home alone or with other people/in an institution).

#### 2.3.2 Other patient characteristics

A nurse also recorded data on each patient’s main diagnosis by using the International Classification of Diseases, 10^th^ Revision, Classification of Mental and Behavioral Disorders (16), and previous seclusion or mechanical restraint measures. History of or current legal charges were also recorded. To complete the study’s questionnaire, a research assistant asked medical staff if the patient was considered resistant to psychotropic treatments, if the patient was under the influence of a psychoactive drug at the time of admission, and whether the patient displayed aggressive and harmful behaviors.

#### 2.3.3 S/R measure

In the structured form, seclusion was defined as either moving the patient to a locked seclusion room or locking up the patient in his or her own room. Restraint was defined as a mechanical restraint, i.e., confining the patient to a restraint bed (17). In France, mechanical restraint can be practiced only in the context of a seclusion measure (6, 7).

We collected the following data for each seclusion and mechanical restraint measure: date, time, main reason for hospitalization and whether the patient was transferred from another department. The duration of S/R was calculated as the difference in days between the start and end date-time. Temporary exits from the isolated room were included in calculating the duration of the event. The main reasons for starting S/R were recorded. Data were extracted from medical records or completed by the medical staff. The research assistant also asked medical staff to describe whether chest belts and other straps were used as part of the mechanical restraint measure.

### 2.4 Analysis

Sociodemographic and medical history characteristics are described for all participants. Categorical variables are described with number (%) and continuous variables with mean (SD).

The following analyses were performed separately for each type of coercive event (seclusion and mechanical restraint). We describe the distribution of the number of events per participant as well as the distribution of the duration of events and the time from admission in the hospital unit to the first event. We used Student *t* test and Pearson correlation to compare the average duration of an event according to patient characteristics. Finally, we performed bivariate logistic regression with the Student *t* test to examine factors associated with the use of an S/R measure versus a seclusion measure (all mechanically restrained participants were also secluded).

## 3 Results

### 3.1 Participant characteristics

During the 3-month recruitment period, 3,274 adults were admitted to participating hospitals: 57.7% male (n=1,889), mean age 43.7 years (SD =17.5), with a main ICD-10 diagnostic code of F20-F29 (schizophrenia, schizotypal, delusional, and other non-mood psychotic disorders) or F30-F39 (mood disorders) in 53.4% (n=1,750) and 22.2% (n=728) of cases, respectively. Admission was voluntary in 44.4% (n=1,455) of patients, while 66.6% (n=1,819) were involuntary: 25.4% patients at the request of a third party (n=832), 12.8% patients at the request of a state representative (n=418) and 17.4% patients for imminent danger (n=569).

**Figure 1:**
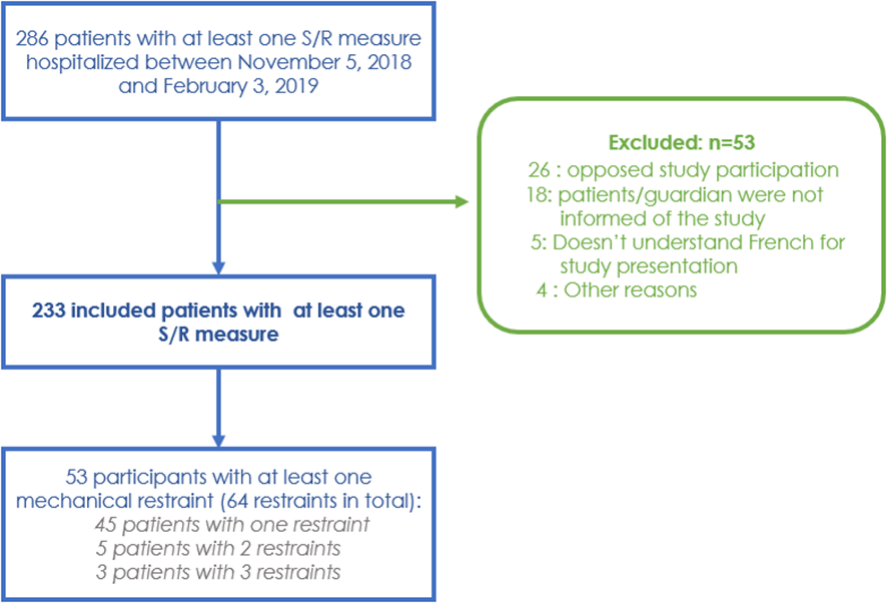
Flow of participants with at least one seclusion or restraint (S/R) measure.

Of these, a total of 286 patients were eligible, and 233 patients were included (**Figure 1**), representing 7.1% of all cases. The main characteristics of the population are shown in **Table 1**. About three quarters of patients (62%) were male. The most common primary diagnosis was psychotic disorders (F20-F29: 66% of participants), followed by mood disorders. Compared to all inpatients, patients subject to seclusion were significantly younger, mean age 38.5 (SD=14.0) vs. 43.7 years (SD=17.5) (p=0.000), and more likely to have a diagnosis of psychotic disorder (F20-F29), 65.7% (n=153) vs. 53.4% (n=1,750), p=0.000. These patients were also more likely to be hospitalized without consent, 91.0% (n=212) vs 66.6% (n=1,819), p=0.000.

**Table 1:** Participant characteristics.

		All participants, n=233	Participants with a mechanical restraint measure n=53
**Sex**	Male	145 (62.2%)	43 (81.1%)
	Female	88 (37.8%)	10 (18.9%)
**Age, years, mean (SD)**		38.5 (14)	33.8 (11)
**Living situation**	Experiencing homelessness	28 (12%)	6 (11.3%)
	Have a residence (including a hotel)	178 (76.4%)	41 (77.4%)
	Institution	20 (8.6%)	4 (7.6%)
	Other	1 (0.4%)	0 (0%)
	Missing	6 (2.6%)	2 (3.8%)
**Mode of commitment in psychiatric care or S/R measure**	Voluntary	21 (9.0%)	4 (7.6%)
	Involuntary commitment at the request of a third party	94 (40.3%)	17 (32.1%)
	Involuntary commitment at the Request of a State representative	64 (27.5%)	22 (41.5%)
	Involuntary commitment in the case of imminent danger	54 (23.2%)	10 (18.9%)
Main diagnosis*	F01-F09: Mental disorders due to known physiological conditions	1 (0.43%)	0
	F10-F19: Mental and behavioral disorders due to psychoactive substance use	5 (2.14%)	2 (3.77%)
	F20-F29: Schizophrenia, schizotypal, delusional, and other non-mood psychotic disorders	153 (65.38%)	37 (69.81%)
	F30-F39: Mood [affective] disorders	51 (21.79%)	7 (13.21%)
	F40-F48: Anxiety, dissociative, stress-related, somatoform and other nonpsychotic mental disorders	2 (0.85%)	0
	F50-F59: Behavioral syndromes associated with physiological disturbances and physical factors	8 (3.42%)	4 (7.55%)
	F60-F69: Disorders of adult personality and behavior	2 (0.85%)	0
	F70-F79: Intellectual disabilities	2 (0.85%)	0
	F80-F89: Pervasive and specific developmental disorders	10 (4.27%)	0
	F90-F98: Behavioral and emotional disorders with onset usually occurring in childhood and adolescence	1 (0.43%)	0
	Unavailable/unspecified	5 (2.14%)	3 (5.66%)
**Main reason for hospitalization**	Anxiety	7 (3%)	1 (1.9%)
	Depressive disorder	6 (2.6%)	1 (1.9%)
	Delusional state	102 (43.8%)	22 (41.5%)
	Mutism, claustration	5 (2.2%)	1 (1.9%)
	Acute intoxication to a substance	2 (0.9%)	(0%)
	Suicidal conduct or risk of suicide	14 (6%)	(0%)
	State of psychomotor arousal	60 (25.8%)	3 (5.7%)
	Dissociative state	10 (4.3%)	15 (28.3%)
	Catatonic state	2 (0.9%)	1 (1.9%)
	Other	25 (10.8%)	9 (17.0%)
**Addiction problem (including alcohol but not tobacco)**	No	135 (57.9%)	31 (58.5%)
	Yes	98 (42.1%)	22 (41.5%)
**Self-harm act/behaviour**	Missing	68 (29.2%)	22 (41.5%)
	No	117 (50.2%)	20 (37.7%)
	Yes	48 (20.6%)	11 (20.8%)
**Aggressive behavior/acts**	Missing	58 (24.9%)	15 (28.3%)
	No	56 (24%)	7 (13.2%)
	Yes	119 (51.1%)	31 (58.5%)
**Has/had legal charges**	Missing	90 (38.6%)	27 (50.9%)
	No	108 (46.4%)	17 (32.1%)
	Yes	35 (15%)	9 (17%)
**Previous mechanical restraint measure**	Missing	79 (33.9%)	19 (35.9%)
	No	88 (37.8%)	11 (20.8%)
	Yes	66 (28.3%)	23 (43.4%)
**Previous seclusion measure**	Missing	58 (24.9%)	18 (34%)
	No	45 (19.3%)	9 (17%)
	Yes	130 (55.8%)	26 (49.1%)
**Considered resistant to psychotropic treatments**	Missing	21 (9%)	5 (9.4%)
	No	169 (72.5%)	36 (67.9%)
	Yes	43 (18.5%)	12 (22.6%)
**Psychoactive treatment at the time of admission**	Missing	14 (6%)	1 (1.9%)
	No	25 (10.7%)	8 (15.1%)
	Yes	126 (54.1%)	27 (50.9%)
	Halted treatment	68 (29.2%)	17 (32.1%)
**Transferred from the emergency department**	No	93 (39.9%)	19 (35.9%)
	Yes	133 (57.1%)	33 (62.3%)
	Missing	7 (3%)	1 (1.9%)
**Referred by another department following the indication for the measure**	No	177 (76%)	39 (73.6%)
	Yes	56 (24%)	14 (26.4%)
**Study center**	Sainte Anne	40 (17.2%)	12 (22.6%)
	Maison Blanche	130 (55.8%)	40 (75.5%)
	Association de Santé Mentale du 13e arrondissement de Paris	44 (18.9%)	1 (1.9%)
	Hopitaux de Saint Maurice	19 (8.2%)	0 (0%)

S/R, seclusion or restraint.

*International Classification of Diseases, 10^th^ Revision, Classification of Mental and Behavioral Disorders.

### 3.2 S/R measures

A total of 281 S/R measures (217 seclusion measures plus 64 mechanical restraints for 53 patients) were recorded. Of the patients included, 206 were first admitted to the participating center after the start of the study, allowing data to be collected on time from admission to S/R events. **Table 2** presents the mean duration of S/R events and the mean time between time from admission to the hospital unit to the first event for the 206 participants who were first admitted after the start of the study. The mean duration of a seclusion measure was 10.2 (SD=14.6) days and a mechanical restraint measure 4.1 (SD=5.6) days. The mean time from admission to an S/R measure was about 3 (SD=6.0) days.

**Table 2:** Mean duration of seclusion and mechanical restraint events (n=206).

	n	Event duration, days mean (SD)
**Mechanical restraint**	64	4.1 (5.6)
**Seclusion**	217	10.2 (14.6)
**Time from admission to restraint**	49	3.1 (6.0)
**Time from admission to seclusion**	167	2.6 (6.2)

Half of the seclusion measures (n=111, 51.2%) were preceded by an alternative: de-escalation talk (n=104, 48.0%) and/or administration of medication (n=84, 38.7%).

In most cases (n=55, 87%), a chest belt and ankle and wrist restraints were used. For 11% (n=7) of cases, these procedures were supplemented with restraining garments. In only one case was the chest belt and ankle restraints used without restraining the wrists.

Most restraint measures took place after a transfer from the emergency department (n=33, 62%) or another department (n=14, 26%).

No adverse effect was reported for 79% (n=42) of participants with a mechanical restraint event. Constipation was the most frequently reported adverse effect (11%, n=6), followed by deep vein thrombosis (2%, n=1) and post-traumatic stress syndrome (2%, n=1).

### 3.3 Factors associated with duration of S/R events

**Table 3** presents the mean duration of an S/R event according to examined variables (bivariate analysis). The longest seclusion measures were for patients with a history of seclusion or restraint measures. Patients with legal charges, those considered resistant to psychotropic treatments, and those with violent behaviors or acts also had a long seclusion duration. No variable was statistically associated with the duration of mechanical restraint measures. Age was not correlated with the duration of a seclusion measure (Pearson correlation coefficient (r) = -0.13, degrees of freedom (df) =51, p=0.35) or duration of a restraint measure (r = -0.01, df=175, p=0.9). The main diagnosis was independent of the duration of seclusion with respectively 10.1 (SD=11.7), 10.4 (SD=14.5) and 13.2 days (SD=14.6) for F20-F29 (Schizophrenia, schizotypal, delusional, and other non-mood psychotic disorders), F30-F39 (Mood [affective] disorders) and other diagnosis, p=0.61.

**Table 3:** Mean duration of seclusion (=217) and mechanical restraint (n=64) measures by patient characteristics.

		Seclusion measures duration			Restraint measures duration		
		N	Mean (days)	p *(t* test)	N	Mean (days)	p *(t* test)
**Sex**	Men	108	11.59	0.13	47	4.62	0.23
	Women	80	8.38		12	2.33	
**Self-harm behavior**	No	98	10.58	0.62	20	3.2	0.93
	Yes	39	9.15		14	3.36	
**Aggression toward other people**	No	48	7.08	0.06	10	1.7	0.2
	Yes	96	11.61		33	3.73	
**Previous seclusion measure**	No	87	6.32	**<.0001**	11	2.09	0.47
	Yes	38	21.03		25	3.24	
**Previous restraint measure**	No	38	5.29	**0.02**	9	2	0.53
	Yes	103	12.31		28	3.07	
**Has/had legal charges**	No	90	7.79	**0.03**	19	2.74	0.83
	Yes	29	14.83		9	3	
**Referred by another department following the indication of the measure**	No	146	10.88	0.25	43	4.58	0.35
	Yes	42	7.95		16	3	
**Patient considered resistant to psychotropic treatments**	No	145	9	**0.04**	40	3.3	0.81
	Yes	27	14.15		14	3	
**Other violent behaviors**	No	56	6.91	**0.03**	11	1.91	0.23
	Yes	75	12.97		24	3.96	

Bold values are significant statistical association.

**Table 4** presents the mean time from admission to an S/R event according to examined variables (bivariate analysis). The only variable associated with a significantly short mean time was referral from another department after the indication of the measure.

**Table 4:** Mean time between admission and seclusion (n=167) or mechanical restraint (n=49) measure according to patient characteristics.

		Time from admission to seclusion			Time from admission to restraint		
		N	Mean (days)	p (*t* test)	N	Mean (days)	p (*t* test)
**Sex**	Men	93	2.53	0.85	40	2.98	0.83
	Women	74	2.72		9	3.44	
**Self-harm behavior**	No	87	2.9	0.98	18	3.72	0.77
	Yes	33	2.94		10	2.9	
**Aggression toward other people**	No	45	2	0.35	7	0.29	0.13
	Yes	80	3.21		28	4.71	
**Previous seclusion measure**	No	36	3.06	0.94	8	2.63	0.85
	Yes	88	2.95		23	2.22	
**Previous restraint measure**	No	72	2.38	0.97	10	2.2	0.94
	Yes	36	2.33		20	2.35	
**Has/had legal charges**	No	80	2.6	0.64	15	3.2	0.63
	Yes	23	1.96		9	4.78	
**Referred by another department after the indication of the measure**	No	**127**	**3.23**	**0.02**	**35**	**4.17**	**0.04**
	Yes	**40**	**0.65**		**14**	**0.29**	
**Patient considered resistant to psychotropic treatments**	No	126	1.81	**<0.001**	34	3.18	0.89
	Yes	26	6.35		10	3.5	
**Other violent behaviors**	No	50	3.02	0.87	8	0.13	0.14
	Yes	63	2.79		20	3.35	

Bold values are significant statistical association.

### 3.4 Factors associated with restraint measures

**Table 5** presents results of bivariate logistic regression examining factors associated with the use of a seclusion plus a mechanical restraint measure versus seclusion alone among participants in only two study centers with seclusion measures: Sainte-Anne Hospital and Maison Blanche (n= 182). Only the sex of the patient was linked to the outcome, with men more likely than women to be mechanically restrained (odds ratio 2.99; 95% confidence interval 1.34–6.65). Also, younger patients were more likely to be mechanically restrained than older patients (33.7% vs 39.7%, p = 0.006).

**Table 5:** Bivariate logistic regression analysis: factors associated with the use of a restraint measure versus the use of a seclusion measure, n=182.

Variable		OR (95% CI)
**Sex (ref=female)**	Male	**2.99 (1.34–6.65)**
**Living situation (ref = experiencing homelessness)**	Have a residence vs experiencing homelessness	1.18 (0.44–3.18)
	Institutionalised vs experiencing homelessness	1.71 (0.37–7.97)
**Mode of commitment in psychiatric care (ref =involuntary commitment in the case of imminent danger)**	Voluntary	1.1 (0.25–4.86)
	Involuntary commitment at the request of a third party	0.98 (0.4–2.4)
	Involuntary commitment at the request of a state representative	2.34 (0.96–5.73)
**Addiction problem (including alcohol but not tobacco) (ref=yes)**	No	1.0 (0.52–1.92)
**Self-harm act/behavior (ref=yes)**	No	0.57 (0.24–1.37)
**Aggressive behavior/acts (ref=yes)**	No	0.43 (0.17–1.07)
**Has/had legal charges (ref=yes)**	No	0.72 (0.28–1.88)
**Previous mechanical restraint measure (ref=yes)**	No	0.52 (0.23–1.21)
**Previous seclusion measure (ref=yes)**	No	1.17 (0.48–2.87)
**Considered resistant to psychotropic treatments (ref=yes)**	No	0.63 (0.28–1.46)
**Psychoactive treatment at the time of admission (ref=halted treatment)**	No	1.49 (0.52–4.31)
	Yes	0.83 (0.4–1.72)
**Transferred from the emergency department (ref=no)**	Yes	1.17 (0.60–2.28)
	Missing	0.91 (0.09–9.31)
**Referred by another department following the indication to perform the measure (ref=yes)**	No	0.71 (0.34–1.5)
**Study center (ref Sainte Anne)**	Maison Blanche	1.0 (0.47–2.15)

OR, odds ratio; 95% CI, 95% confidence interval.

Bold values are significant statistical association.

## 4 Discussion

Our study describes the characteristics of S/R measures among patients in four Parisian psychiatric centers. We found a relatively high overall prevalence (7%) of such measures in the participating hospitals, which is in line with previous studies (18–22). Seclusion was mainly used in young patients (23, 24) with a primary diagnosis of schizophrenia, schizotypal and delusional disorders (F20-F29) (23–25), admitted without their consent (23), after transfer from the emergency department and with few reported adverse events.

S/R measures were particularly observed in involuntary admissions: it can be assumed that a reduction in involuntary admissions could lead to a reduction in S/R measures. A recent study suggests that the implementation of advance directives in psychiatry significantly reduces involuntary admissions (26). We have started to use this tool in our health structures. In addition, the physical environment of the ward has been shown to have an impact on the use of seclusion and restraint (27): our structures are beginning to be equipped with “sensory rooms”, a way of limiting tension and avoiding S/R (9, 28, 29).

Furthermore, S/R measurements occurred mainly in patients transferred from another department, especially the emergency department. This observation is partly due to patients arriving in an acute state with behavioral problems, often requiring the intervention of emergency teams (fire, emergency medical service) or the police. In addition, patients are usually transferred from another department due to lack of space or lack of adapted and appropriate rooms or equipment or trained staff.

The duration of a seclusion measure was about 10 days, comparable with recent French findings (30) less than in Japan (15) or the Netherlands (31), but much longer than in most countries (17, 32, 33). However, different definitions, inconsistent registration procedures and different data collection methods limit comparisons (31). In France, until 2022, the duration of each episode of seclusion was recorded without taking into account temporary exits: the duration may therefore be overestimated. The new way of recording the event in the French medico-economic databases will allow a more precise description of each event (6). The duration of seclusion was often longer for patients considered to be resistant to psychotropic treatments than for others. In contrast to other studies (12, 15, 30), primary diagnosis was not associated with the duration of seclusion. We found no effect of gender (30). The mean time from admission to the hospitalization unit and the S/R measure was the shortest for patients who were referred from another department after the indication of the measure. Male sex and younger age were associated with experiencing a restraint measure, which agrees with the literature (11).

In France, the average length of stay in seclusion rooms was 15 days in 2014 (34) and 12 days in 2017 (35). Our study may indicate a significant reduction in seclusion duration. However, more efforts are needed to reduce the duration of S/R measures, which are shorter in other countries: about 8 hours in Germany (33) and 4 hours in an Australian study (36).

### 4.1 Limitations

The incidents of S/R measures may have been underreported by the medical staff because of social desirability bias. Also, some S/R measures may not have been reported due to recall bias or the unavailability of the medical staff involved in the measures. However, by analyzing the medico-economic data (RIMP, the French psychiatric DRGs), we found 291 patients with a seclusion measure at the time of the study, which allowed us to consider our data to be fairly exhaustive. Moreover, incomplete data would affect the prevalence of S/R measures, not their duration, or associated factors.

## 5 Conclusions

Despite rigorous recommendations and legislation limiting the use and duration of S/R measures, we describe prolonged durations of such measures for psychiatric patients in Paris. Our findings could be used to identify opportunities to reduce the occurrence and duration of S/R measures in general psychiatry, such as emergency department-specific protocols.

## Data availability statement

The raw data supporting the conclusions of this article will be made available by the authors, without undue reservation.

## Ethics statement

The studies involving humans were approved by French medical research ethics committee (September 6, 2018: Committee of Persons Protections of the Parisian region IV, no. IRB 00003835). The studies were conducted in accordance with the local legislation and institutional requirements. Written informed consent for participation was not required from the participants or the participants’ legal guardians/next of kin in accordance with the national legislation and institutional requirements.

## Author contributions

V-LM: Formal analysis, Methodology, Writing – review & editing. F-KL: Formal analysis, Writing – original draft. JL: Investigation, Writing – review & editing, Data curation. CL: Conceptualization, Writing – review & editing. AC: Conceptualization, Writing – review & editing. CB: Conceptualization, Writing – review & editing. JM: Visualization, Writing – review & editing. XB: Investigation, Writing – review & editing. FP: Data curation, Methodology, Project administration, Writing – review & editing. M-NV: Conceptualization, Writing – review & editing.
